# Successful dabigatran reversal after subdural hemorrhage using idarucizumab in a mobile stroke unit: A case report

**DOI:** 10.1097/MD.0000000000020200

**Published:** 2020-05-22

**Authors:** Sibi Thirunavukkarasu, Hayrapet Kalashyan, Glen Jickling, Thomas Joseph Jeerakathil, Harsha Kamble Jayaprakash, Brian H. Buck, Ashfaq Shuaib, Ken Butcher

**Affiliations:** Division of Neurology, University of Alberta Hospital, Alberta, Canada.

**Keywords:** antibodies, anticoagulant agents, dabigatran, hematoma subdural, humanized, Idarucizumab, monoclonal

## Abstract

**Rationale:**

Idarucizumab is a specific reversal agent for patients with bleeding related to the anticoagulant dabigatran. There are no prior descriptions of Idarucizumab administration in the prehospital setting for intracranial hemorrhage.

**Patient concerns:**

An 82-year-old woman treated with dabigatran for atrial fibrillation developed acute focal weakness. This led to activation of emergency medical services and assessment in the mobile stroke unit (MSU).

**Diagnosis:**

Computed tomography of the brain performed in the MSU revealed an acute subdural hematoma.

**Interventions:**

The patient was treated with Idarucizumab in the MSU.

**Outcomes:**

The subdural hematoma was treated with a burr hole evacuation and the patient was discharged to a rehabilitation facility without residual focal neurological deficits.

**Lessons:**

Idarucizumab can be used safely and effectively to treat dabigatran-associated intracranial hemorrhage in the prehospital setting.

## 1 Introduction

Idarucizumab is a specific reversal agent for patients with bleeding related to the anticoagulant dabigatran.^[1]^ Current guidelines recommend reversal of anticoagulation in patients acutely bleeding at critical sites including the brain, spinal cord, and pericardium.^[2]^ There is evidence that anticoagulant-associated intracerebral hematomas expand more frequently, with subsequent neurological deterioration and poor functional outcome, than in patients not taking anticoagulants.^[3,4,5]^ Rapid reversal of the coagulopathy is therefore recommended.^[6]^ Although there are sparse data related to subdural hemorrhage expansion in anticoagulated patients, the principle of rapid reversal is also applicable.^[7]^

Idarucizumab has been used to treat dabigatran-associated intracranial hemorrhage acutely in hospital.^[8]^ There are currently no reports of reversal of dabigatran related bleeding in the pre-hospital setting. We are currently evaluating the utility of a mobile stroke unit (MSU) in a rural setting.^[9]^ Following approval of Idarucizumab, this drug was placed on the MSU. We describe Idarucizumab administration in the MSU after diagnosis of a dabigatran-related subdural hemorrhage (SDH).

## 2 Case summary

An 82-year-old, right-handed woman, was assessed as part of the AmbulanCe Housed Ischemic Stroke trEatment with intraVEnous Thrombolysis (ACHIEVE) study.^[10,11]^ The study was approved by University of Alberta Health Research Ethics Board; ID - Pro00037601. The patient provided informed consent for publication of this report.

The patient was taking dabigatran 110 mg, twice daily for atrial fibrillation (AF), on the day of assessment, she developed sudden onset left sided facial droop and dysarthria. As part of the ACHIEVE study she was assessed in the MSU, which is equipped with a computed tomography (CT) scanner (Ceretom).

**Figure 1:**
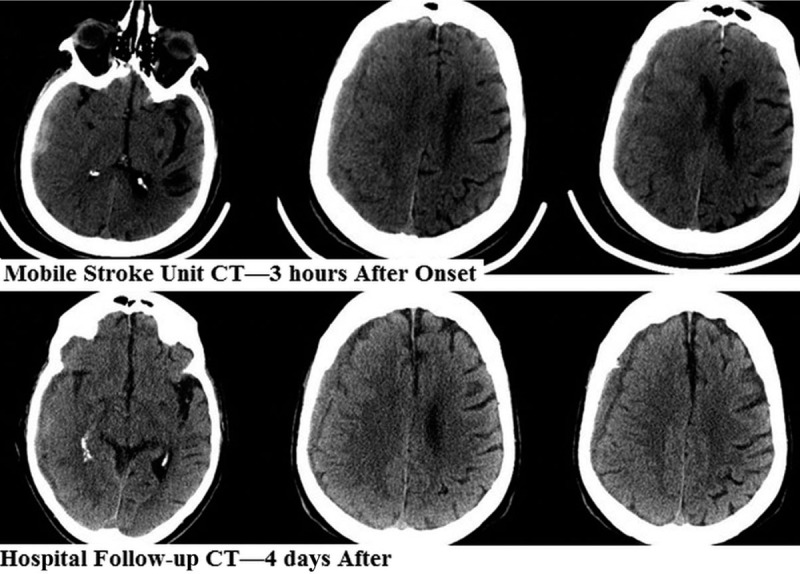
Computed tomography scan performed pre-Idarucizumab treatment in the mobile stroke unit demonstrating acute on chronic right subdural hemorrhage. Post-treatment scan in hospital demonstrates stability of the hemorrhage.

The past medical history was significant for coronary artery bypass grafting, and a fall 2 weeks prior to symptom onset. The patient confirmed taking dabigatran on the day of symptom onset. Examination in the MSU 1 hour after symptom onset revealed upper motor neuron pattern left facial weakness and moderate dysarthria. Mobile CT scan demonstrated a right subdural hematoma (Fig. 1). Point of care testing for dabigatran levels was not available on the MSU.

The patient was treated with Idarucizumab 5 g intravenously in the MSU. Thrombin time (TT) and partial thromboplastin time (PTT) after arrival at hospital were 16.6 seconds (Normal range 14.3–19.7) and 25 seconds (Normal 27–38) respectively. The patient was admitted to the neurosurgery service and managed conservatively. Repeat CT scan 72 hours later demonstrated a stable subdural hematoma (Fig. 1). A burr hole evacuation procedure was performed on day 4. Following burr hole trephination, the patient was discharged to a rehabilitation facility. At the time of discharge, the patient did not have any focal neurological deficits.

## 3 Discussion

Idarucizumab is a specific reversal agent for the anticoagulant dabigatran that has been shown to normalize clotting indices including TT, in patients presenting with bleeding or requiring urgent surgery.^[1,8,12]^ The treatment priority in anticoagulant-associated intracranial bleeding is reversal of the coagulopathy and attenuation of hematoma expansion. Hematoma expansion tends to occur early and therefore rapid reversal is recommended.^[13,14]^

Although a pre-treatment TT/aPTT were not available on the MSU, the patient did confirm taking dabigatran the day of symptom onset, suggesting she was therapeutically anticoagulated at the time of assessment. The normal post-treatment TT and stability of the subdural hematoma on CT scans suggests successful treatment in our patient.

Although the primary aim of pre-hospital stroke programs is thrombolysis for ischemic stroke, the mobile CT also facilitates the early diagnosis of intracranial hemorrhage and rapid reversal in anticoagulant associated cases. For this reason, the MSU carries prothrombin concentrate complex for vitamin K antagonist related intracranial bleeding.^[13,14]^ Idarucizumab was placed on the MSU soon after approval for use in Canada. Currently, there is no specific reversal agent for patients taking factor Xa antagonists including rivaroxaban, apixaban and edoxaban. Patients with Xa antagonist related bleeding are currently treated empirically with prothrombin complex concentrate (PCC) as per treatment guidelines.^[6]^

Idarucizumab can also be used to reverse the effects of dabigatran in patients presenting with ischemic stroke symptoms in order to facilitate thrombolysis.^[15,16]^ In fact, the only previous report of Idarucizumab use for stroke in the pre-hospital setting was for this purpose, reflecting the fact that ischemic stroke remains more common than intracranial bleeding in patients with atrial fibrillation who are anticoagulated.^[17]^

Summary: Idarucizumab can be used safely and effectively to treat dabigatran-associated intracranial hemorrhage in the pre-hospital setting. We suggest mobile units carry reversal agents, including Idarucizumab, prothrombin complex concentrate and where approved/available, andexanet alpha for management of Xa antagonist associated bleeding.^[18,19]^

## Author contributions

**Investigation:** Hayrapet Kalashyan.

**Methodology:** Hayrapet Kalashyan, Glen Jickling, Ken Butcher.

**Resources:** Sibi Thirunavukkarasu.

**Supervision:** Ashfaq Shuaib, Ken Butcher.

**Validation:** Sibi Thirunavukkarasu, Ken Butcher.

**Writing – original draft:** Sibi Thirunavukkarasu, Hayrapet Kalashyan, Glen Jickling, Thomas Joseph Jeerakathil, Harsha Kamble Jayaprakash, Brian Hilton Buck, Ashfaq Shuaib, Ken Butcher.

**Writing – review & editing:** Sibi Thirunavukkarasu, Hayrapet Kalashyan, Glen Jickling, Thomas Joseph Jeerakathil, Brian Hilton Buck, Ken Butcher.
